# History of Obstinate Hiccough Cured by Colchicum

**Published:** 1816-12

**Authors:** Thomas Raven


*For the London Medical and Physical Journal.*

### *History of obstinate Hiccough cured by Colchicum;* by Mr. Thomas Raven.

ANN CULPET, æt. 19, of a florid complexion, full habit of body, and, to all appearance, in exceedingly good health, had been a morning patient of Dr. Alderson, on account of an almost incessant hiccup, for many weeks.

As the disorder had not abated in all that time, the Doctor took her into the hospital, on Saturday, Feb. 13, 1816, and discharged her cured April 6, 1816; but not till after she had been largely and repeatedly blooded, had been blistered still oftener, and rubbed with the Cerat. ant. tart. till numberless large pustules had been induced—put twice a-week into the warm bath—and had taken Tinct. digital., liquor. antimon. tartarizat., solutio fowler. mineral., ferrum, hydrargyrus, and other medicines. A few weeks after she had been discharged, the complaint returned with redoubled violence. She was again put on the list of patients, and was going over her former course of medicines, when the Doctor, observing the benefit his patients had derived from the Colchicum in chorea, resolved to try its virtues in this patient. Accordingly, she was ordered to take xxx♏. of the tincture three times a-day, when, to our astonishment, in six days, the hiccup was entirely removed. She continued the medicine for a short time; and was then discharged cured. The Doctor is now giving it in those very troublesome disorders denominated hypochondriacal, of which we see a great number in this hospital.

As you have been so flattering to me as to admit a few of my rude sketches, I may possibly take the liberty of troubling you with more.

*Norwich; Nov.* 11, 1816.

---

**[We are always thankful for well-authenticated practical facts; and none can be more important than remedies successful in obstinate diseases from causes not easily ascertained.—Edit.]**

